# Season Match Loads of a Portuguese Under-23 Soccer Team: Differences between Different Starting Statuses throughout the Season and Specific Periods within the Season Using Global Positioning Systems

**DOI:** 10.3390/s22176379

**Published:** 2022-08-24

**Authors:** João Barreira, Fábio Y. Nakamura, Ricardo Ferreira, João Pereira, Rodrigo Aquino, Pedro Figueiredo

**Affiliations:** 1 Research Center in Sports Sciences, Health Sciences and Human Development (CIDESD), University of Maia, 4475-690 Maia, Portugal; 2 Departamento de Apoio ao Rendimento, Vitória Sport Clube, 4810-914 Guimaraes, Portugal; 3 LabSport, Department of Sports, Center of Physical Education and Sports (CEFD), Federal University of Espírito Santo, Vitória 29075810, Brazil; 4 Physical Education Department, College of Education, United Arab Emirates University, Al Ain P.O. Box 15551, Abu Dhabi, United Arab Emirates; 5 Portugal Football School, Portuguese Football Federation, 4711-852 Oeiras, Portugal; 6 Research Center in Sports Sciences, Health Sciences and Human Development (CIDESD), 5001-801 Vila Real, Portugal

**Keywords:** global positioning systems, football, performance, match load

## Abstract

This study aimed to quantify the external match loads (EMLs) of a Portuguese u-23 soccer team, competing at the highest national level for the age group, comparing players with different starting status throughout a competitive season and specific blocks. Thirty-five outfield soccer players were split into three groups for the entire season analysis and for each 3-month block, based on the percentage of games played as a starter. The three groups consisted of “starters” (≥55% of the games as a starter), “fringe” (30–54%), and “non-starters” (<30%). EMLs were recorded using 10 Hz GPS technology throughout the whole season (26 matches). Differences (*p* < 0.05) were found for total distance (TD), exposure time, and the number of accelerations and decelerations between starters and non-starters throughout the season (*d* = 0.73 to 1.08), and within each block (*d* = 0.59 to 1.68). Differences were also found between starters and fringe players for the number of accelerations in Block 2 (*p* = 0.03; *d* = 0.69), and TD (*p* = 0.006; *d* = 1) and exposure time (*p* = 0.006; *d* = 0.95) in Block 3. Differences in the EML were almost always accompanied by large differences in game time. Our results highlight the differences in the EML of starters and non-starters, emphasizing the need for compensatory training, especially with players that obtain significantly less playing time, to prepare the players for match demands (e.g., high-intensity efforts such as sprinting, accelerations, and decelerations).

## 1. Introduction

Soccer is a team-based sport played at various competitive levels (e.g., junior, senior, amateur, professional, and elite) with an emphasis on intermittent movements such as brief bouts of high-intensity actions and long periods of low-intensity exercise [1]. Extensive research has shown that senior players typically cover a total distance (TD) of 10–14 km [2,3,4,5] and perform 150–250 intense actions per match [6]. These high-intensity actions, such as accelerations and decelerations, as well as sprints, are considered one of the most important activities in the match, being associated with the most frequent actions in goal situations [7,8]. Of note, similar external match loads (EML) have been reported between an English professional under-23 team and the club’s first team [5], evidencing that young adults already perform similarly to seniors.

Therefore, to understand the physical demands of the sport, accurate and objective quantification of the actions performed by the players is needed [9,10,11,12]. Global Positioning System (GPS) devices have grown in popularity over the years and are seemingly more practical than traditional time–motion analysis [10]. The adoption of these devices to track players’ movements enables minimal human involvement and faster data collection and analysis [13]. GPS devices can have an acquisition frequency of 1, 5, 10, and 15 Hz. Accordingly, a GPS with a sampling frequency of 1 Hz, for example, might be incapable of accurately registering movements that take less than a second to complete [14]. The growing use of these devices and the emergence of new brands in the market have also made them more accessible across the different competitive levels. In Portugal, a new national under-23 championship was recently created to support the competitive transition from youth to senior level. Typically, a team roster in this championship comprises almost 40 players from various age categories. Accordingly, a relatively high rotation of players and starters throughout the season is expected. Maintaining squad fitness throughout the season is challenging in this scenario when almost half the roster is not participating in the match [15,16]. In fact, Anderson et al. [15] previously highlighted the differences, especially in high-intensity actions and match exposure, between starters and non-starters, which have important implications for training planning and load managing.

This fact has gained relevance as the match can be considered the most demanding session of the week [17]. Of note, it has already been reported that physical fitness can decrease in non-starter players during the season due to a lack of exposure to competition [18,19]. For that reason, compensatory training sessions, on the match-day or following the match-day, have been adopted for non-starters or players that were not even on the bench [20]. Consequently, supporting decisions related to match/training exposure based on objective measures is essential to help coaching staff with the programming of training and exercises [21]. However, to date, no study has analyzed the magnitude of longitudinal differences in EML measures between different starting statuses on a competitive under-23 soccer team.

Therefore, this study aimed to quantify the EML using GPS devices across a competitive soccer season in national-level Portuguese players with different starting statuses and compare the differences between these players throughout the whole season and in distinct blocks, as well as the EML of players between the different blocks of the season. We hypothesized that players with fewer starting appearances would present a lower EML than players with more games played as “starters” [15] and that a high player rotation throughout the season would result in a considerably steady EML across the blocks [22].

## 2. Materials and Methods

### 2.1. Subjects

Thirty-five outfield soccer players (mean ± SD: age 19.7 ± 1.2 years old, body mass 73.7 ± 6.1 kg, height 181.7 ± 7.1 cm) from a Portuguese under-23 team, competing at the highest national level for the age group, participated in this study. The data were collected as part of the team routines in which players are monitored across the full season. This was approved by the Federal University of Espírito Santo Ethics Committee (5.292.105). The study was conducted according to the Declaration of Helsinki.

### 2.2. Study Design

**Figure 1:**
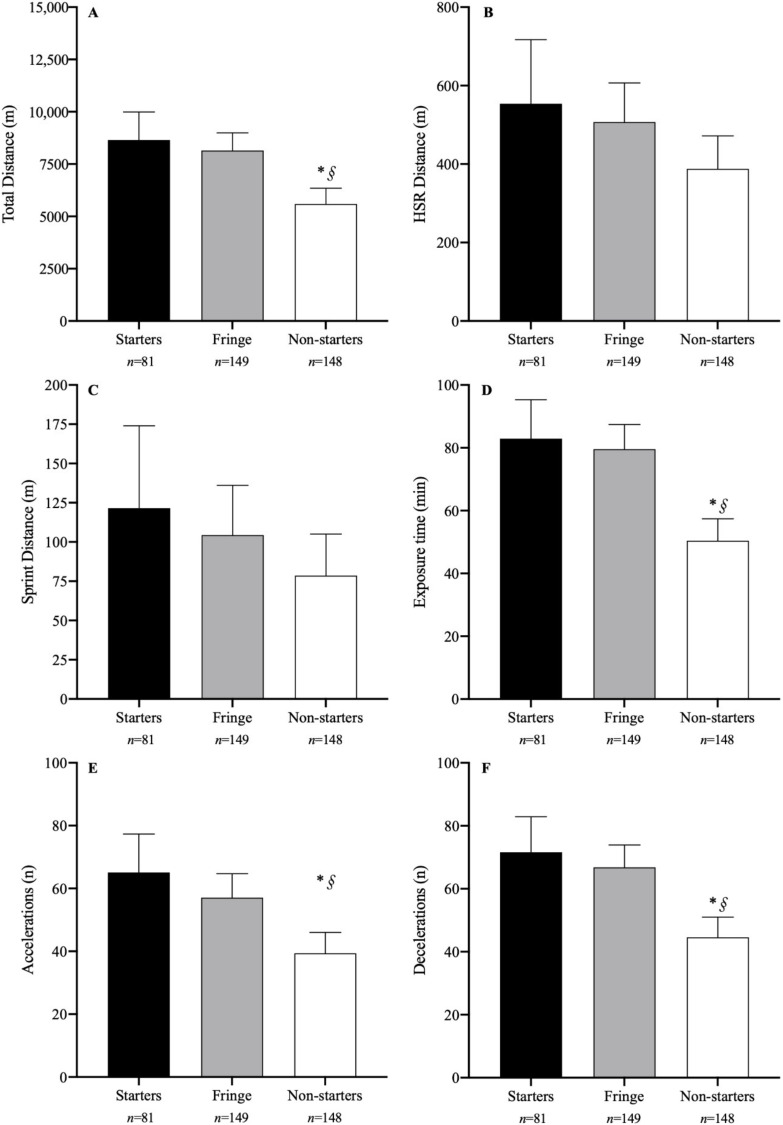
Mean ± 95% IC estimates for total distance (**A**), HSR distance (**B**), sprint distance (**C**), exposure time (**D**), and number of accelerations (**E**) and decelerations (**F**) throughout the whole competitive season. * significantly different from starters; § significantly different from fringe. The number of observations (*n*) for each condition is presented below the respective group. Abbreviations: HSR—high-speed running.

**Figure 2:**
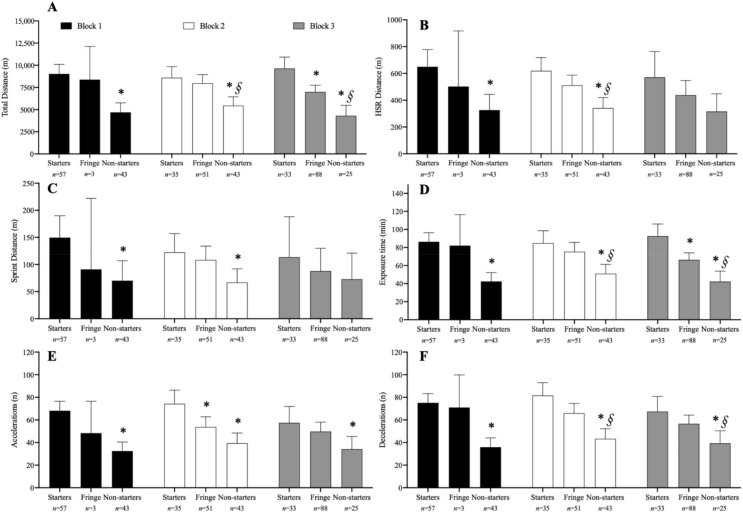
Mean ± 95% IC estimates for total distance (**A**), HSR distance (**B**), sprint distance (**C**), exposure time (**D**), and number of accelerations (**E**) and decelerations (**F**) throughout the first block (black bars), second block (white bars), and third block (grey bars). * significantly different from starters; § significantly different from fringe. The number of observations (*n*) for each condition is presented below the respective group. Abbreviations: HSR—high-speed running.

This was a longitudinal observational study. Information regarding the layout of the season and the different blocks is presented in Table 1. Data were collected throughout a 35-week (August to April) period during the 2021/2022 competitive season. The team that participated in the analysis competed in two distinct competitions, which took place consecutively during the season (Table 1). A total of 26 matches were analyzed across the competitions (Championship phase 1, North Group, *n* = 12; Cup Qualifying, North Group, *n* = 14). The season was analyzed as a whole and further divided into three blocks of three months each. Players were split into three groups for the entire season analysis and according to their playing status in each block. The three groups consisted of “starters”, “fringe”, and “non-starters”, and were split based on the percentage of games played as a starter for the entire season and during each block. Starting players started ≥55% of the games, fringe players started 30–54% of the games, and non-starters started <30% of the games, as adapted from Anderson et al. [15]. The number of observations for each condition is presented in Figure 1 and Figure 2. All games were played on natural grass pitches, as required by the competition rules (competition regulations can be assessed online at www.fpf.pt/Competições/Futebol-Masculino/Liga-Revelação (accessed on 10 July 2022), in Portuguese).

### 2.3. External Match Load

External match loads were recorded using 10 Hz Global Positioning Systems (GPS) technology (Apex GPS, STATSports, Newry, North Ireland, UK), which had been previously validated [23]. The devices were fitted to each player’s upper back (i.e., between the scapulae), as the manufacturer recommends. They provide data on the time and location of satellite tracking devices and receive information that determines the signal traffic. The Apex GPS 10 Hz has shown a distance bias of 1.05 ± 0.87%, 2.3 ± 1.1%, and 1.11 ± 0.99% in the 400 m trial, 128.5 m circuit, and 20 m trial, respectively, and a V_peak_ bias of 2.36 ± 1.67% [23].

EML variables included exposure time (the total amount of time a player participated in the game), total distance, high-speed running (HSR) distance (≥19.8 km/h), sprint distance (≥25.2 km/h), and the number of accelerations (>2 m/s^2^) and decelerations (<−2 m/s^2^) [24].

### 2.4. Statistical Analysis

Data are presented as mean ± 95% CI, unless otherwise stated. The Kolmogorov–Smirnov distribution test was performed to check the data distribution. A linear mixed-model analysis was performed to test the differences in the EML variables between the different starting status groups. Starting status was included as a fixed effect and player ID as a random effect. To assess the differences in EML across blocks for each starting status, one-way ANOVAs for independent samples were used. An α-level of 0.05 was set as the significance level for statistical comparisons. When significance was reached, post hoc pairwise comparison tests using Bonferroni correction were computed to assess differences between the groups. The t and F statistics were converted to effect size (ES) [25]. Effect sizes were calculated using Cohen’s *d* (d) and the magnitude of the ES was interpreted as follows: *trivial* < 0.2; *small* > 0.2; *moderate* < 0.6; *large* < 1.2; *very large* > 2.0 [26]. Statistical analyses were conducted using R statistical software (version 4.0.2, R Foundation for Statistical Computing, Vienna, Austria) with the lme4 [27], emmeans [28], and effectsize [29] packages.

## 3. Results

### 3.1. Entire Season Analysis

A total of 35 players played at least one game throughout the season (mean ± SD 10.8 ± 6.5 games played per player), and no player participated in all games. Thirty-two players started in at least one game throughout the season (7.5 ± 5.0 games as a starter per player). Comparisons for each EML variable between starters, fringe, and non-starters throughout the season are presented in Figure 1. *Moderate* differences between starters and non-starters were found for TD (*p* = 0.001; *d* = 0.71), exposure time (*p* = 0.003; *d* = 1.02), and the number of accelerations (*p* = 0.002; *d* = 0.8) and decelerations (*p* = 0.001; *d* = 0.93). Non-starters also covered less TD (*p* = 0.001; *d* = 1.06), had less exposure time (<0.001; *d* = 1.08), and a lower number of accelerations (*p* = 0.003; *d* = 0.68) and decelerations (*p* = 0.001; *d* = 0.9) than fringe players. Even though starters tended to present higher EML values than fringe players throughout the season, these differences were not significant (*p* < 0.05).

### 3.2. Within-Blocks Analysis

Twenty-three players participated in at least one game during the first block of the season (4.5 ± 2.1 games played per player), thirty players during the second block (4.3 ± 2.3 games played per player), and twenty-nine during the third and last block (5.1 ± 3.2 games played per player). Six players participated in all games during the first block, two players during the third, and no player was involved in all matches of the second block. No single player was considered a “starter” across all blocks. Comparisons for each EML variable between starters, fringe, and non-starters throughout each block of the season are presented in Figure 2.

In Block 1, *moderate* to *large* differences were found for TD (*p* < 0.001; *d* = 1.41), HSR distance (*p* = 0.003; *d* = 0.89), sprint distance (*p* = 0.02; *d* = 0.69), exposure time (*p* = 0.001; *d* = 1.54), and number of accelerations (*p* < 0.001; *d* = 1.53) and decelerations (*p* = 0.001; *d* = 1.68) between non-starters and starters.

In Block 2, *moderate* differences were found between starters and fringe players for the number of accelerations (*p* = 0.03; *d* = 0.69). In addition, *small* to *large* differences between starters and non-starters were found for TD (*p* = 0.001; *d* = 0.95), HSR (*p* = 0.001; *d* = 1.06), sprint distance (*p* = 0.04; *d* = 0.59), exposure time (*p* < 0.001; *d* = 0.92), and number of accelerations (*p* < 0.001; *d* = 1.08) and decelerations (*p* < 0.001; *d* = 1.28). There were also *small* to *moderate* differences (*p* < 0.05; *d* = 0.46 to 0.73) between fringe and non-starters for all EML variables, except for the number of accelerations.

In Block 3, starters covered more TD than fringe players (9628 vs. 7000 m, *p* = 0.006; *d* = 1, *moderate*) and had more exposure time (93 vs. 66 min, *p* = 0.006; *d* = 0.95, *moderate*). *Moderate* to *large* (*d* = 0.53 to 1.23) differences between starters and non-starters were found for TD (*p* < 0.001; *d* = 1.26), exposure time (*p* < 0.001; *d* = 1.19), and number of accelerations (*p* = 0.01; *d* = 0.53) and decelerations (*p* = 0.007; *d* = 0.69). There were also *small* to *moderate* (*d* = 0.43 to 0.62) differences between fringe and non-starters for TD (*p* = 0.001; *d* = 0.62), exposure time (*p* = 0.003; *d* = 0.57), and number of decelerations (*p* = 0.04; *d* = 0.43).

### 3.3. Between-Blocks Analysis

Multiple comparisons revealed *small* to *moderate* differences between Block 1 and 3 (*p* = 0.05; *d* = 0.52) and Block 2 and 3 (*p* = 0.005; *d* = 0.78) in the number of accelerations performed by starters, with Block 2 presenting the highest number (74) and Block 3 the lowest number (58) of accelerations. There were also fewer decelerations performed by starters on Block 3, compared with Block 2 (82 vs. 67, *p* = 0.01; *d* = 0.7, *moderate*). Similar values for the other EML variables (*p* < 0.05) were found per starting status.

## 4. Discussion

The present study aimed to quantify the EML of under-23 professional soccer players with different starting status throughout a competitive season, using 10 Hz GPS units. Our findings reveal that players with starting status covered significantly more distance, had more exposure time, and performed more accelerations and decelerations than non-starters throughout the season and in each block. On the other hand, contrary to our hypothesis, there were not many differences between starters and fringe players for the EML, with a few differences arising only in the analysis by blocks.

To the authors’ knowledge, this is the first study to analyze the longitudinal differences in EML in a Portuguese under-23 professional soccer team. Previously, Anderson et al. [15] quantified the cumulative training and match load of an English Premier League team during the whole competitive season using GPS devices and Prozone^®^. The authors found no differences between starting status (starters vs. fringe vs. non-starters) for the cumulative TD and exposure time (training + matches), but reported differences in high-speed actions. Nevertheless, comparisons between studies should be made carefully, as the methodologies used are very different, since we used a longitudinal approach with linear mixed models (i.e., models that allow for missing data and the inclusion of random effects) [30], while Anderson et al. [15] used ANOVAs for the analysis, which do not consider within-subject variation and cannot handle missing data, which is common in longitudinal research [31].

During the competitive season, soccer players participate in a high number of matches, which usually leads to them being under prolonged physiological stress (functional and oxidative) that affects the immune system, and which may cause hormonal-related imbalances [2,18,32,33]. When analyzing the EML, starters always displayed *moderate* to *largely* (*d* = 0.69 to 1.68) higher values for TD, exposure time, and number of accelerations and decelerations than non-starters. Obvious differences for physical and physiological demands between training and matches exist [34,35]; nonetheless, such data could suggest that the long-term physiological adaptations arising from match exposure within these groups could differ [15]. Therefore, coaches and support staff may need to adopt specific strategies to ensure that the players receive similar weekly external load exposure and are ready to cope with the match demands. Players with less or no game time will require at least one training session to compensate for the match loads [20]. However, replicating the EML is somewhat difficult and dependent on various factors, such as the compensatory training mode, the playing position, the player, the team playing style, and others [36].

Different strategies can be implemented, such as high-intensity interval training (HIIT), small-sided games (SSG), or a mixture of both, with the aim of preparing the athletes for the demands of the match [37]. Running-based drills, such as HIIT, can elevate HSR and sprint distance exposure, compared to SSG [37]. However, a higher number of acceleration and deceleration actions can be obtained with SSG [37]. Thus, future research should explore how these two strategies can be mixed and explore if it might be viable to use them in top-up sessions with players with less or no match exposure. Nevertheless, in the current literature, it has been suggested that the training load of the compensatory session might not be enough to compensate for low or nonexistent match exposure [16]. For instance, the study by Stevens et al. [20] showed that non-starters’ compensatory training revealed significantly lower values (e.g., lower TD, and a lower number of accelerations and decelerations) than those obtained by starters during the match. On the other hand, Martín-García et al. [38] reported that the external loads on MD + 1C (the compensatory training session following the match) for players without game time were high, corresponding to over 50% (TD, HSR, accelerations, and decelerations) of the match values, although not developing HSR and sprinting qualities in these players. Nevertheless, for the starters, the match is the most demanding session of the week, leaving non-starters with considerably lower training values [20], which can lead to physical fitness decrements [39], and a general risk of underloaded non-starters sustaining an injury [40]. Making efforts to reduce the differences in the workloads between players with distinct levels of match participation could help avoid under- or overloading players [41].

Contrary to our hypothesis, starters and fringe players had a similar EML. Thirty-five outfield players were used throughout the season, resulting in a considerably high player rotation. Throughout the season, the mean number of games as a starter for starters and fringe players was 16.3 ± 1.1 and 10.5 ± 1.8 games, respectively. Considering that 26 games were played throughout the season, this is a relatively low number for each group. Consequently, this high player rotation throughout the season might explain the minimal differences in EML between blocks. It is known that, as the competitive season progresses, fatigue accumulation can be a problem [42], especially with players that play and start in most games of the season. In our study, only the number of accelerations (*d* = 0.52 and 0.78, Block 1 vs. 3 and Block 2 vs. Block 3, respectively) and decelerations (*d* = 0.70, Block 2 vs. Block 3) decreased in Block 3 among the starters, compared with the previous two blocks, which might be attributed to the number of players utilized and consequent squad rotation. In fact, squad rotation and player availability are important factors when coping with the high demands of soccer training and matches, contributing to overall physical performance and team results [22,43].

When analyzing the season in blocks, and specifically in Block 3, starters covered more TD than fringe players (*d* = 1.00) and presented higher exposure time (*d* = 0.95), which may be a consequence of lower match participation by fringe players (8 ± 0.7 vs. 4.2 ± 0.9 games, out of 10). An increase in the number of fringe observations throughout the blocks (Figure 2) also needs to be pointed out. Interestingly, Block 3 presented the greatest differences between the two groups. In line with what was previously reported by Anderson et al. [15], these differences also reflect the differences in exposure time, with starters obtaining more game time and, consequently, higher EML values. It should be noted that Block 3 was the final block of the season, with fewer players utilized compared with the other phases, which could, in part, explain some reductions in the EML, even though they were small. This block involved the cup qualifying stage and a month with six official games, the highest number of games the team played throughout the season. For this reason, once again, it is necessary to implement compensatory sessions or have the players competing in a different team (e.g., B team or under-19), whenever possible [20].

Despite the relevance of the findings presented, this study has limitations. The lack of data from training sessions leaves us with no information regarding the possible compensatory strategies adopted by the coaching and support staff. Since most of the players in this study could participate in other games (i.e., under-19 or B team), having access to GPS data from these games would help understand how players’ match exposure was managed throughout the season and if playing in a lower/higher team would deliver the same EML stimulus as playing for the under-23 s. Moreover, match-related contextual factors, such as match location, outcome, and opposition, which can influence physical performance [44], were not controlled in this research. Furthermore, the sample size for each group varied in each block, with some low sample sizes (i.e., fringe players in block 1; *n* = 3) affecting the analysis. Thus, in the future, research might mitigate these limitations by increasing the sample size (i.e., more than one season or more teams included) and including data from training and compensatory sessions.

### Practical Applications

Our results may have important implications for training programming since significant differences were found for high-intensity actions such as accelerations and decelerations, specifically between starters and non-starters. As mentioned above, coaches and support staff might want to adopt different compensatory training strategies for these players so that, when called to play, they are ready to cope with match demands. These strategies can vary from analytic drills, such as HIIT and change-of-direction drills, to soccer-specific ones, such as SSG and large-sided games. However, organizing friendly matches for these players or allowing them to play for another team within the club (i.e., B team) could possibly be a good option due to the specific demands imposed by the match that cannot be easily replicated in training.

## 5. Conclusions

In summary, we quantified the EML of players with different starting statuses throughout a competitive national-level Portuguese under-23 soccer season. The findings of the present study highlight that throughout the whole season and within the different blocks analyzed, starters present *moderate* to *large* differences in TD, exposure time, number of accelerations and decelerations, compared with non-starters. Our results reinforce the need for compensatory training to prepare the players for the match demands (e.g., high-intensity efforts such as sprinting, accelerations, and decelerations) whenever a player does not participate in a full match or play the match at all. In addition, few differences in EML were detected across the competitive blocks, being restricted to the starting group that reduced accelerations and decelerations toward the end of the competition.

## Figures and Tables

**Table 1:** The layout of the 2021/2022 competitive soccer season analyzed.

Year	2021					2022			
Competition	Championship (Matches, *n* = 12)					Cup Qualifying (Matches, *n* = 14)			
Block	1 (Matches, *n* = 7)			2 (Matches, *n* = 9)			3 (Matches, *n* = 10)		
**Month**	August	September	October	November	December	January	February	March	April
**No. Games**	2	2	3	2	3	4	6	3	1

## Data Availability

Data are not publicly available due to restrictions from the club where the data were obtained and will be shared upon specific request.
